# Dietary supplementation with *Epimedium* contributes to the improvement of hormone levels, gut microbiota, and serum metabolite composition in the Chinese forest musk deer (*Moschus berezovskii*)

**DOI:** 10.3389/fvets.2024.1497115

**Published:** 2025-01-22

**Authors:** Shan Xie, Qinlin Yang, Zaixiang Ying, Mingcheng Cai, Wenqiao Fan, Hanyu Gao, Xiaolan Feng, Yongjiang Wu

**Affiliations:** ^1^College of Smart Agriculture, Chongqing University of Arts and Sciences, Yongchuan, China; ^2^College of Biology and Food Engineering, Chongqing Three Gorges University, Wanzhou, China; ^3^Chongqing Institute of Medicinal Plant Cultivation, Nanchuan, China

**Keywords:** *Epimedium*, *Moschus berezovskii*, hormone levels, gut microbiota, serum metabolism

## Abstract

The Chinese forest musk deer (*Moschus berezovskii*) is a small ruminant animal with special economic value. It is listed as a National Level I key protected species in China. However, these animals are prone to stress responses in captive environments. *Epimedium*, a traditional Chinese herb with aphrodisiac and anti-stress properties, may have potential benefits for the health of the captive Chinese forest musk deer, though its efficacy requires further investigation. This study aimed to evaluate the effects of dietary supplementation with *Epimedium* on the hormone levels, gut microbiota composition, and serum metabolism of the Chinese forest musk deer. The fourteen adult male Chinese forest musk deer with similar initial body weights (7.0 ± 0.3 kg) and an average age of 4.5 years were randomly divided into two groups, each containing seven animals. The control group was fed a standard diet without *Epimedium*, while the *Epimedium* group received the standard diet supplemented with 15 g *Epimedium* /kg DM. The results indicated that the inclusion of *Epimedium* in the diet increased dry matter intake (DMI) and improved the ratio of feed to gain (F/G), with an increase in fecal testosterone levels (*p* < 0.05). 16S rDNA sequencing analysis revealed that *Epimedium* enhanced the richness and diversity of the gut microbiota in the Chinese forest musk deer, increasing the relative abundance of beneficial bacteria such as Firmicutes, while reducing the relative abundance of the potentially pathogenic Proteobacteria (*p* < 0.05). A widely targeted metabolomics analysis identified 25 differential metabolites between the two groups. Significant alterations were observed in key metabolic pathways related to lipid metabolism, hormone regulation, and antioxidation, such as ovarian steroidogenesis, tyrosine metabolism, and glycerophospholipid metabolism. Furthermore, correlation analysis between gut microbiota and serum differential metabolites showed that the relative abundances of *Clostridia_vadinBB60_group* and *UCG-010* were positively correlated with anserine and 7-ketocholesterol, respectively (*p* < 0.05). In conclusion, *Epimedium* positively influenced feed intake and hormone levels in the Chinese forest musk deer by modulating gut microbiota composition and serum metabolism.

## 1 Introduction

The Chinese forest musk deer (*Moschus berezovskii*), also known as the dwarf musk deer, is a rare and endangered species primarily found in the mountain forests of Sichuan, Gansu, and Shaanxi provinces in China (1). This animal is highly valued for its unique musk gland, which secretes musk, a substance of significant economic and medicinal importance in fields such as medicine, perfumery, and cosmetics (2, 3). However, the artificial breeding of the Chinese forest musk deer is fraught with considerable challenges. Due to their inherently timid and solitary nature (4), it is particularly difficult to meet their growth and reproductive requirements in captive environments (5). This adversity directly compromises the health of the Chinese forest musk deer and the quality of the musk they produce (6, 7). Compared to their wild counterparts, the captive Chinese forest musk deer are more susceptible to various health issues, including diarrhea, pneumonia, and malnutrition (8, 9). Therefore, the conservation of this rare species and the enhancement of its health status are of paramount importance.

Nutritional supplementation is a critical method for improving animal health and survival capabilities (10). For wildlife, appropriate nutritional interventions can significantly enhance physiological functions and increase resistance to adverse environmental conditions. *Epimedium*, a traditional Chinese medicinal herb, is extensively utilized in traditional Chinese medicine (TCM) due to its significant bioactive properties, including anti-inflammatory, antioxidant, and hormone-regulating effects. It is particularly recognized for its potential to modulate sexual function and enhance physical strength (11–13). For the Chinese forest musk deer, hormonal levels are critical indicators of health and reproductive capacity (14). Abnormal hormonal levels can result in reproductive disorders, delayed growth and development, and other health issues (15). The primary bioactive compounds in *Epimedium*, particularly icariin, are believed to effectively promote testosterone and sperm production, thereby improving reproductive function and overall health status (16). Additionally, flavanols extracted from *Epimedium* have been shown to enhance sexual function by stimulating Leydig cells in the testes, leading to increased secretion of testosterone and other androgens (17).

The gut microbiota is an essential component of animal health, playing critical roles in nutrient absorption, immune regulation, and disease prevention (18, 19). Flavonoids and other bioactive compounds in *Epimedium* improve digestive absorption and immune status by modulating the composition and function of the gut microbiota (20). Incorporating *Epimedium* into broiler diets can modulate the abundance of beneficial bacteria, such as *Lactobacillus*, improve gut microbiota composition, and increase the concentration of metabolites like lactic acid and short-chain fatty acids, thereby enhancing metabolic function (21). *Epimedium* can also improve the health status of rats by regulating lipid metabolism, energy metabolism, and amino acid metabolism. This helps to reduce the levels of harmful metabolites in the blood while increasing the levels of beneficial metabolites (22). Additionally, the flavonoids in *Epimedium* can enhance the activity of antioxidant enzymes such as superoxide dismutase (SOD) and glutathione peroxidase (GSH-Px) (23). These enzymes play a crucial role in scavenging free radicals and protecting cells from oxidative damage. These characteristics make *Epimedium* a promising natural feed additive, suitable for use in livestock and poultry farming, offering an effective solution for improving animal health and economic efficiency.

In view of this, the present study aims to explore the potential effects of *Epimedium* as a dietary supplement on the gut microbiota composition, serum metabolic components, and hormone levels in the Chinese forest musk deer. The results could provide new scientific evidence for enhancing the health and hormonal regulation of the captive Chinese forest musk deer.

## 2 Materials and methods

### 2.1 Experimental design

The experiment was conducted at the Chinese forest musk deer breeding base of the Institute of Medicinal Plant Cultivation in Chongqing, China. The fourteen adult male Chinese forest musk deer with similar initial body weights (7.0 ± 0.3 kg) and an average age of 4.5 years were randomly divided into two groups, each containing seven animals. The CK group was fed a standard diet without the addition of *Epimedium*, while the EPI group received the standard diet supplemented with 15 g *Epimedium* /kg DM. The composition and nutritional levels of the standard feed are shown in Table 1. All the Chinese forest musk deer were housed individually, and the environmental conditions, including temperature, humidity, and management methods, were kept identical for both groups. One week before the trial, the ventilation equipment was thoroughly inspected to ensure proper functioning, and the pens were meticulously cleaned and disinfected. Daily management during the trial period was conducted according to the protocol, with feeding scheduled at 3:00 PM each day. The pre-trial period lasted 7 d, followed by a formal trial period of 30 d.

**Table 1:** Composition and nutrient levels of the basal diet (DM basis) %.

Items	Content
Ingredients	
Leaves of *Pittosporum truncatum Pritz*	50.00
Corn	30.00
Soybean meal	11.80
wheat bran	3.50
Corn germ meal	2.40
NaCl	0.30
CaHPO_4_	0.50
Limestone	0.50
Premix^a^	1.00
Total	100.00
Nutrient levels	
Dry matter	73.50
Metabolic energy/(MJ /kg)^b^	8.24
Crude protein	10.64
Neutral detergent fiber	30.23
Acid detergent fiber	15.26
Calcium	0.57
Phosphorus	0.31

a The premix provided the following per kg of the diet: VA 7000 IU, VD 1800 IU, VE 40 IU, Cu 12 mg, Fe 60 mg, Mn 50 mg, Zn 40 mg, I 1.0 mg, Se 0.27 mg, Co 0.3 mg.

b Metabolic energy is the calculated value, while the rest are measured values.

### 2.2 Sample collection

During the trial period, the remaining feed was weighed daily using an electronic scale before feeding to calculate the dry matter intake (DMI). Given that the Chinese forest musk deer are classified as a national first-grade protected animal in China, they are highly sensitive to external environments and prone to stress reactions, which can even lead to death. Growth performance and serum metabolomics analyses require direct manipulation of the animals, including procedures such as weighing and blood collection, which can induce stress in the Chinese forest musk deer. Therefore, in this experiment, we selected only the three Chinese forest musk deer per group for these operations to minimize potential impacts on other individuals and ensure the smooth progression and safety of the experiment. On days 1 and 30 of the trial, the three randomly selected Chinese forest musk deer from each group were weighed after a 12 h fasting period to determine the average daily gain (ADG). On the final day of the trial, the three Chinese forest musk deer from each group were randomly selected for blood collection. The blood samples were placed in coagulation-promoting tubes and centrifuged at 3000 rpm for 10 min at 4°C to collect the serum. All serum samples were stored at −80°C for subsequent analysis. On the same day, fresh fecal samples were collected from all the 14 Chinese forest musk deer, placed in sterile centrifuge tubes, and rapidly frozen in liquid nitrogen. These samples were then stored at −80°C for 16S rDNA sequencing of the gut microbiome.

### 2.3 Fecal hormone level analysis

The levels of testosterone, estradiol, and progesterone in the fecal were measured using enzyme-linked immunosorbent assay (ELISA) kits. These kits were purchased from Quanzhou Ruixin Biotechnology Co., Ltd.

### 2.4 Fecal microbiota profiling

The total DNA of fecal bacteria was extracted using the E.Z.N.A. Soil DNA Kit (Omega Bio-tek, Inc., United States). Concentration and quality of the genomic DNA were checked by NanoDrop 2000 spectrophotometer (Thermo Scientific Inc., United States). The V3–V4 regions of the bacterial 16S rDNA gene were amplified using universal primers 338F (5’-ACTCCTACGGGA-GGCAGCAG-3′) and 806R (5’-GGACTACHVGGGTWTCTAAT-3′). The universal primers with barcode sequences were synthesized and the amplification was carried out on an ABI 9700 PCR instrument (Applied Biosystems, Inc., United States). After purifying the PCR amplification products, the concentration was measured, and high-throughput sequencing was performed on the Illumina Novaseq sequencing platform.

Sequencing data were processed using Pear software (24) (version 0.9.6) for sequence assembly, filtering, and chimera removal, resulting in optimized sequences. High-quality sequences were clustered into OTUs using Vsearch software (25) (version 2.7.1) with a sequence similarity threshold of 99%. Species classification for each OTU was determined by aligning the sequences with the Silva138 database (26) using the BLAST algorithm (27). Alpha and Beta diversity analyses were performed based on OTU and abundance data using QIIME2 software (28) (version 2024.2). Species composition bar plots were generated using R project (29) (version 3.6.0) based on species annotation and relative abundance results. LEfSe analysis was conducted using Python software (version 2.7) (30). The sequencing was conducted at Beijing Allwegene Technology Co., Ltd.

### 2.5 Determination of serum metabolites

The sample stored at −80°C refrigerator was thawed on ice and vortexed for 10 s. 50 μL of serum sample and 300 μL of extraction solution (ACN: Methanol = 1:4, V/V) containing internal standards were added into a 2 mL microcentrifuge tube. The sample was vortexed for 3 min and then centrifuged at 12000 rpm for 10 min (4°C). 200 μL of the supernatant was collected and placed in −20°C for 30 min, and then centrifuged at 12000 rpm for 3 min (4°C). A 180 μL aliquots of supernatant were transferred for LC–MS analysis. The relative concentrations of serum metabolites were analyzed using an LC-ESI-MS/MS system (UPLC, ExionLC AD, https://sciex.com.cn/; MS, QTRAP^®^ System, https://sciex.com/). After the analysis, the raw data obtained were imported into Progenesis QI software (31) (version 3.0) for data preprocessing, which was used for subsequent analysis.

Metabolite information was obtained by annotating the database using HMDB^1^ and Metlin.^2^ Partial least squares discriminant analysis (PLS-DA) was performed using R project. For two-group analysis, differential metabolites were determined by Variable Importance in Projection (VIP > 1) and *p*-value (*p*-value <0.05, *t*-test). Identified metabolites were annotated using the KEGG Compound database^3^, and the annotated metabolites were then mapped to the KEGG Pathway database.^4^ The analysis was conducted at Beijing Allwegene Technology Co., Ltd.

### 2.6 Correlation analysis between rumen microbiota and serum metabolite profiles

Correlation analysis was performed between differential microbiota and metabolites. The spearman correlation coefficients between microbiome and metabolite data were calculated using the psych package in the R project. Correlation heatmaps were generated using the heatmap package.

### 2.7 Statistical analysis

All data were analyzed using independent sample *t*-tests, and the results are presented as mean ± standard error of the mean (SEM). Experimental data with *p* < 0.05 were considered statistically significant.

## 3 Results

### 3.1 The effects of *Epimedium* on growth performance and hormone levels in the Chinese forest musk deer

As shown in Table 2, compared to the CK group, the final BW and ADG tended to increase in the EPI group (*p* < 0.1). The DMI and F/G in the EPI group were higher than those in the CK group (*p* < 0.05), indicating that dietary supplementation with *Epimedium* significantly increased the feed intake and feed conversion ratio in the Chinese forest musk deer. As shown in Table 3, *Epimedium* supplementation increased fecal testosterone levels in the Chinese forest musk deer (*p* < 0.05). Compared to the CK group, the progesterone levels in the fecal samples of the EPI group showed a trend towards increase (*p* < 0.1). Although there were slight increases in estradiol levels, these changes were not statistically significant. This suggests that *Epimedium* can effectively regulate hormone levels in the Chinese forest musk deer.

**Table 2:** Growth performance of the Chinese forest musk deer.

Item	CK	EPI	*p*-value
Initial BW, kg	6.91 ± 0.24	7.31 ± 0.53	0.685
Final BW, kg	7.15 ± 0.18	7.91 ± 0.29	0.089
ADG, kg	0.09 ± 0.06	0.02 ± 0.01	0.090
DMI, g/d	99.48 ± 3.13	110.55 ± 2.86	0.012
F/G	1.70 ± 0.26	0.74 ± 0.05	0.024

DMI, dry matter intake; Initial BW, initial body weight; Final BW, final body weight; ADG, average daily gain; F/G, the ratio of feed to gain; EPI, *Epimedium* group; CK, control group; The statistical results are shown as means ± SEM.

**Table 3:** Fecal hormone levels in the Chinese forest musk deer.

Item	CK	EPI	*p*-value
Progesterone, ng/mL	5.37 ± 0.30	6.29 ± 0.16	0.055
Estradiol, pg./g	892.51.68 ± 51.04	1012.16 ± 58.95	0.200
Testosterone, ng/g	2.57 ± 0.29	3.68 ± 0.21	0.037

EPI, *Epimedium* group; CK, control group; The statistical results are shown as means ± SEM.

### 3.2 Gut microbiome 16S rDNA sequencing analysis

#### 3.2.1 The effects of *Epimedium* on the abundance and diversity of gut microbiota

**Figure 1:**
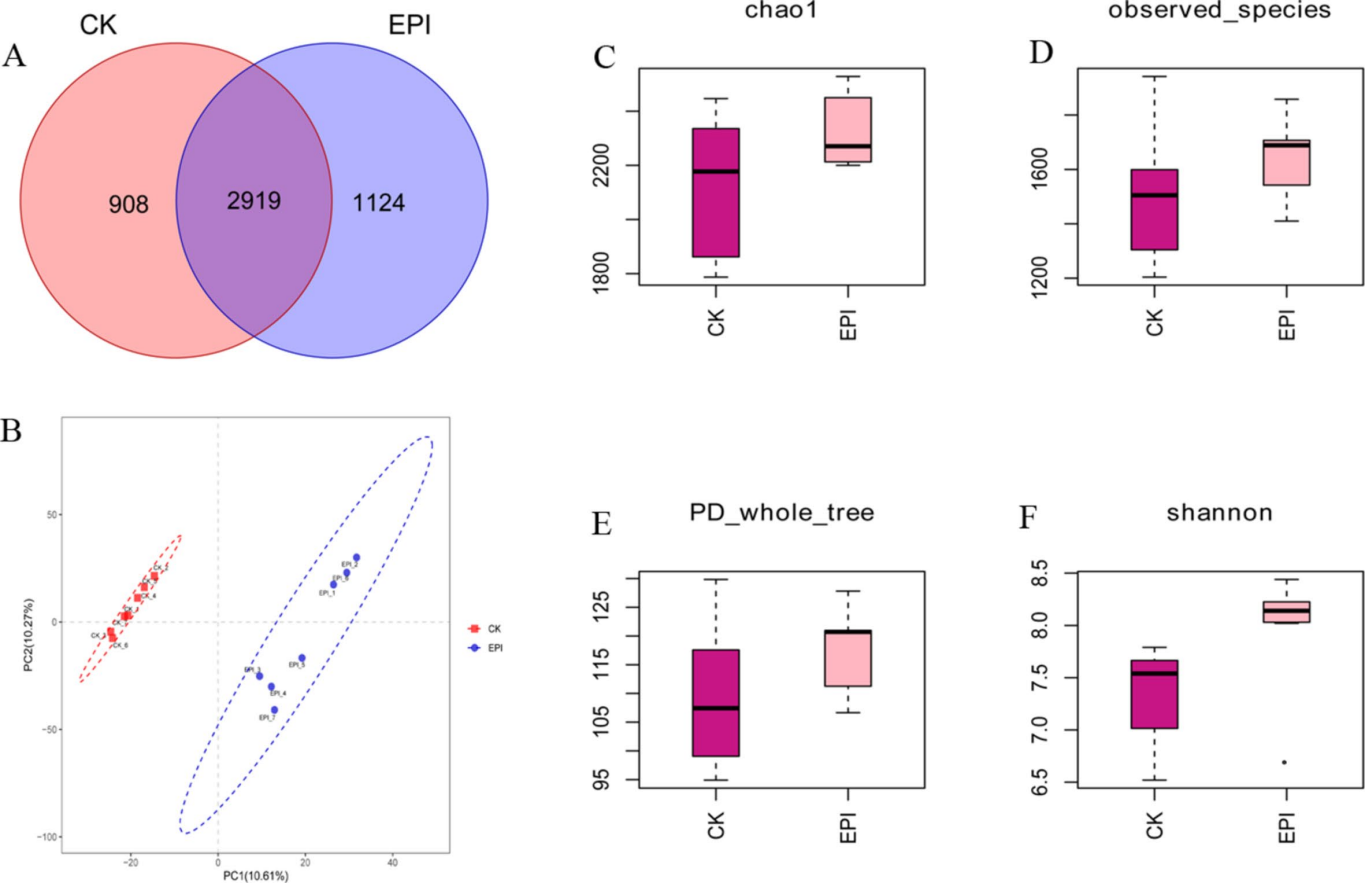
Comparison of gut microbial diversity between the two groups. The Venn diagram shows the number of shared or unique OTUs **(A)**. PLS-DA analysis based on OTUs **(B)**. Box plot of Chao1 index **(C)**. Box plot of observed species index **(D)**. Box plot of PD_whole_tree index **(E)**. Box plot of Shannon index **(F)**. EPI, *Epimedium* group; CK, control group.

As shown in Figure 1A, the EPI group had 1,124 unique operational taxonomic units (OTUs), while the CK group had 908 unique OTUs. Both groups shared 2,919 OTUs. The alpha diversity indices of 14 samples were calculated using the QIIME2 software (Supplementary Table S1). Compared to the CK group, the Chao1 and Observed species indices of the EPI group were slightly higher, but the differences were not statistically significant (Figures 1C,D). Additionally, the Shannon index in the EPI group showed a trend towards increased values, although the change in the PD_whole_tree index was not significant (Figures 1E,F). Beta diversity analysis revealed that the samples from the CK and EPI groups tended to cluster internally, with distinct separation between the groups, indicating significant differences in the gut microbiota composition between the two groups (Figure 1B).

#### 3.2.2 The effects of *Epimedium* on the gut microbiota composition

**Figure 2:**
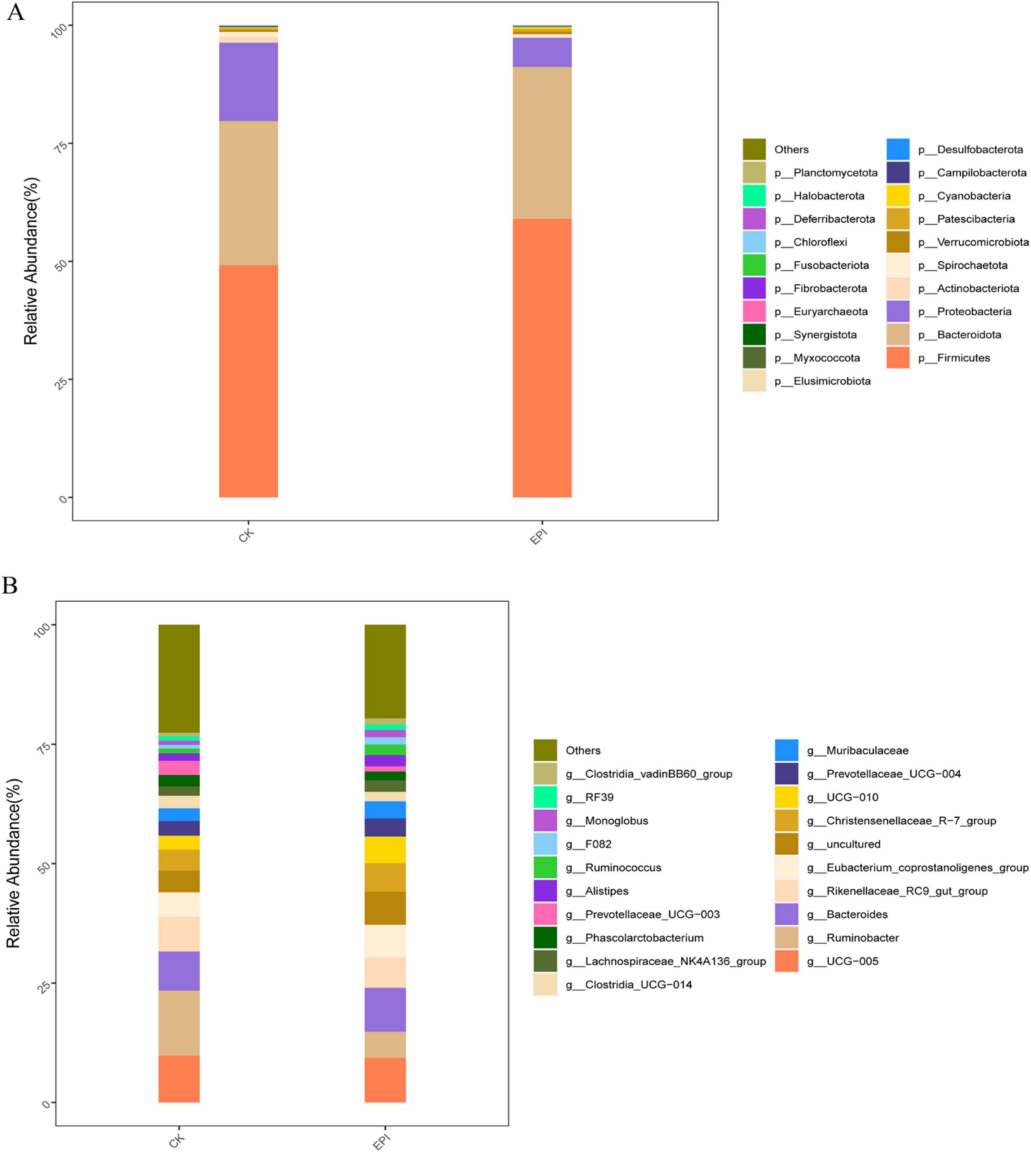
Effects of *Epimedium* on gut microbial community. Phylum−level **(A)**. Genus−level **(B)**. EPI, *Epimedium* group; CK, control group.

A total of 21 phylum, 33 class, 68 order, 117 family, and 258 genus were identified across both groups. As shown in Figure 2A, at the phylum level, the gut microbiota of the Chinese forest musk deer was primarily composed of Bacteroidetes, Firmicutes, and Proteobacteria, with Firmicutes being the dominant phylum. After dietary supplementation with *Epimedium*, the relative abundance of Firmicutes in the EPI group was higher compared to that in the control group, while the relative abundance of Proteobacteria was lower in the EPI group than in the control group (*p* < 0.05). Notably, Firmicutes are considered beneficial bacteria, whereas Proteobacteria are viewed as potential pathogens, indicating that *Epimedium* supplementation may have improved the gut microbiota composition of the Chinese forest musk deer. At the genus level, the relative abundance of *Ruminobacter* and *UCG-005* decreased, while the relative abundance of the beneficial genus *Bacteroides* increased in the EPI group compared to the CK group (Figure 2B).

#### 3.2.3 Gut microbiota differential species analysis

**Figure 3:**
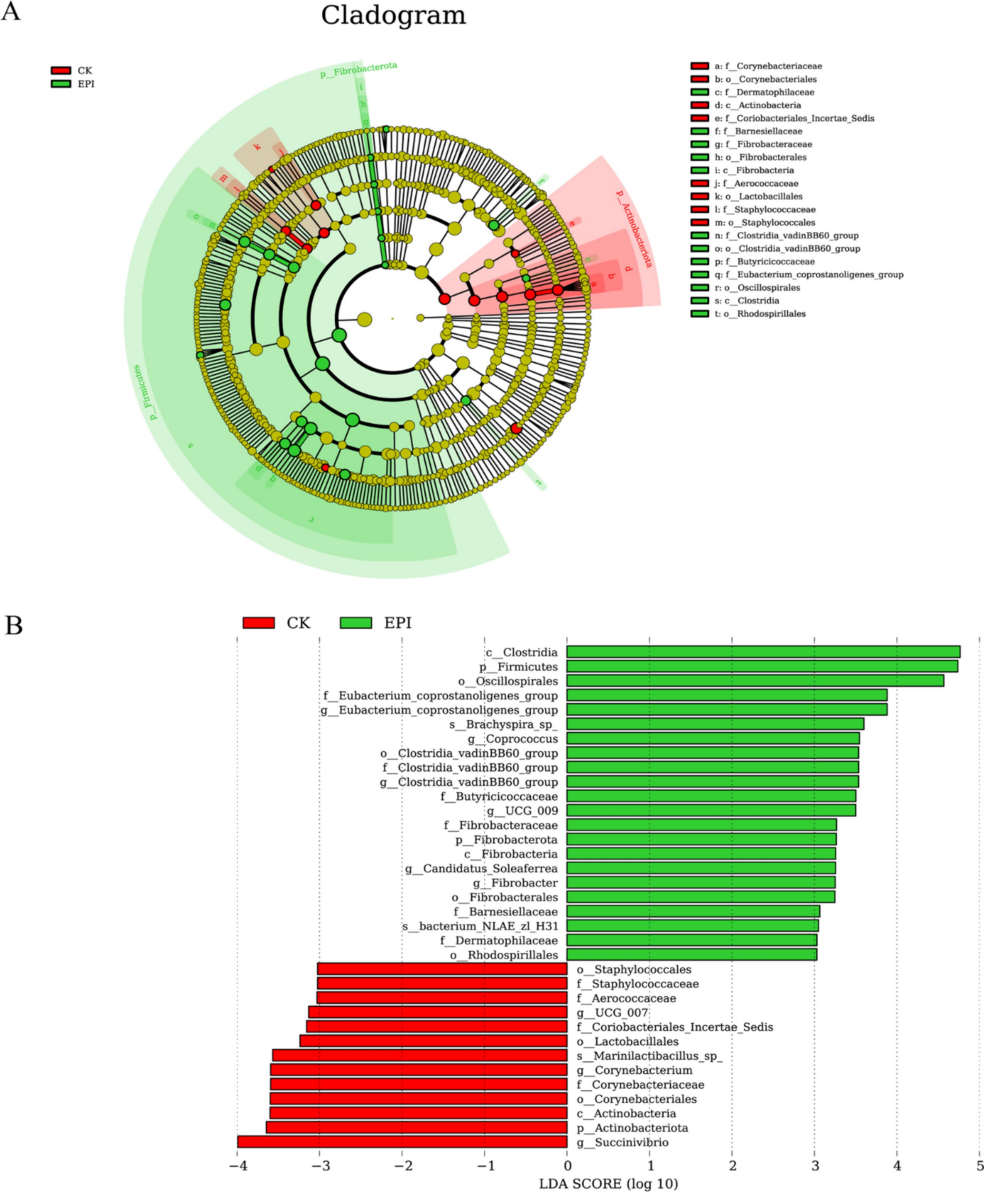
LEfSe cladogram comparing microbial communities between two groups **(A)**. The red represents CK, and the green represents EPI. Significantly different bacterial taxa identified by the linear discriminant analysis effect size **(B)**. Bacteria with an LDA score > 3 are considered to be significantly discriminative. The red represents CK, and the green represents EPI. CK, control group; EPI, *Epimedium* group.

Linear discriminant analysis Effect Size (LEfSe) analysis revealed significantly dominant species in both groups. As shown in Figure 3A, 8 clades were found to have higher abundances in the CK group, while 12 clades were more abundant in the EPI group. The differences in the abundances of various microbial taxa between the CK and EPI groups are depicted in Figure 3B. In the CK group, the most significant difference was observed for the genus *Succinivibrio*, whereas in the EPI group, the classes *Clostridia* and Firmicutes, as well as the order *Oscillospirales*, exhibited notable differences. Among these, *Clostridia* and Firmicutes showed the greatest intergroup differences, with LDA scores greater than 4. Firmicutes is a critical component of the normal gut microbiota, playing a significant role in maintaining intestinal health and helping to prevent diarrhea and other gastrointestinal diseases.

#### 3.2.4 Prediction of gut microbial functions

**Figure 4:**
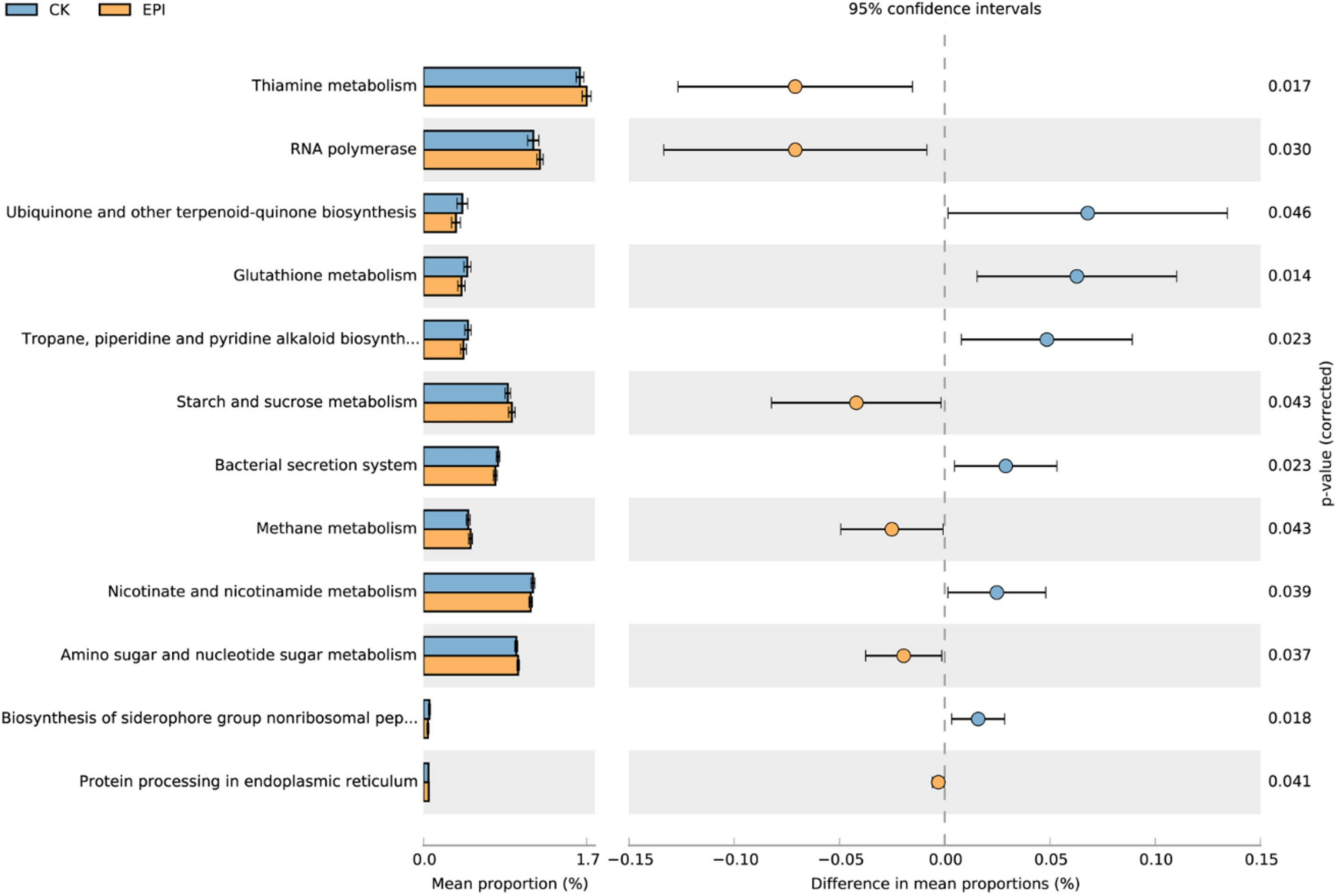
Microbial functional analysis. The blue represents CK, and the yellow represents EPI. CK, control group; EPI, *Epimedium* group.

Prediction of the metabolic functions of the gut microbiota revealed a relatively high abundance of functional genes associated with thiamine metabolism, nicotinate and nicotinamide metabolism, amino sugar and nucleotide sugar metabolism, and RNA polymerase (Figure 4). Differential analysis using a *t*-test identified 12 pathways with significant differences in related genes between the two groups. Compared to the CK group, the EPI group exhibited enhanced pathways in gut microbiota, including ubiquinone and other terpenoid-quinone biosynthesis, glutathione metabolism, and nicotinate and nicotinamide metabolism (*p* < 0.05). Conversely, pathways such as thiamine metabolism, starch and sucrose metabolism, and methane metabolism were significantly reduced in the EPI group (*p* < 0.05). The functional gene classification of the gut microbiota primarily involved energy metabolism, antioxidant protection, and DNA repair.

### 3.3 Serum widely targeted metabolomics analysis

#### 3.3.1 Serum metabolite multivariate statistical analysis

**Figure 5:**
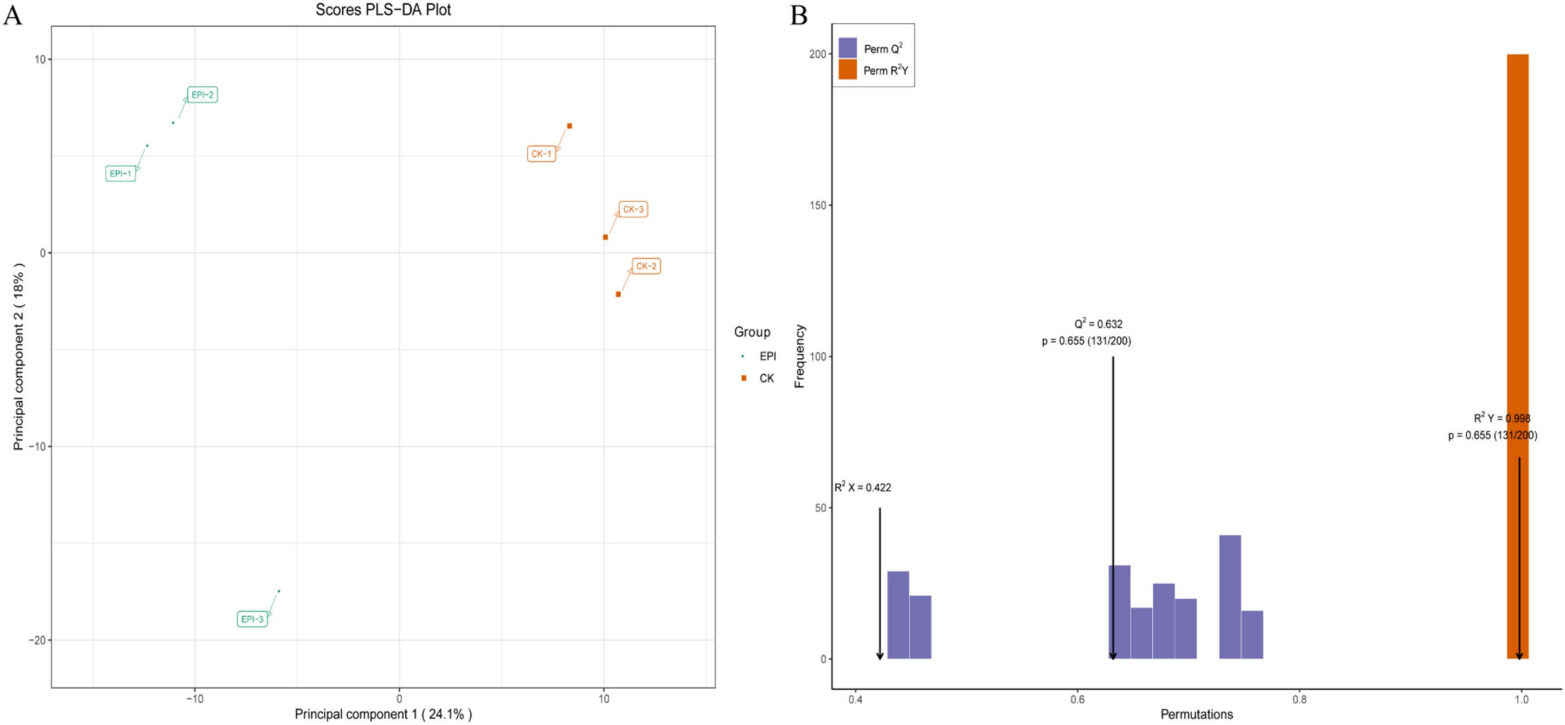
PLS-DA score plot for the CK and EPI groups **(A)**. The yellow represents CK, and the green represents EPI. PLS-DA model validation plot **(B)**. CK, control group; EPI, *Epimedium* group.

As shown in Figure 5A, there is a clear separation of metabolites between the two experimental groups, with a well-defined clustering pattern, indicating significant differences between the metabolites of the EPI group and those of the CK group. To assess the presence of overfitting in the PLS-DA model, permutation tests were conducted for statistical validation of the PLS-DA model. R^2^Y and Q^2^ represent the explanatory and predictive abilities within the PLS-DA model, respectively, with values closer to 1 indicating greater stability and reliability of the model. With R^2^Y = 0.998 and Q^2^ = 0.632, the model demonstrates good predictive ability and reliable results (Figure 5B).

#### 3.3.2 Serum differential metabolites

**Figure 6:**
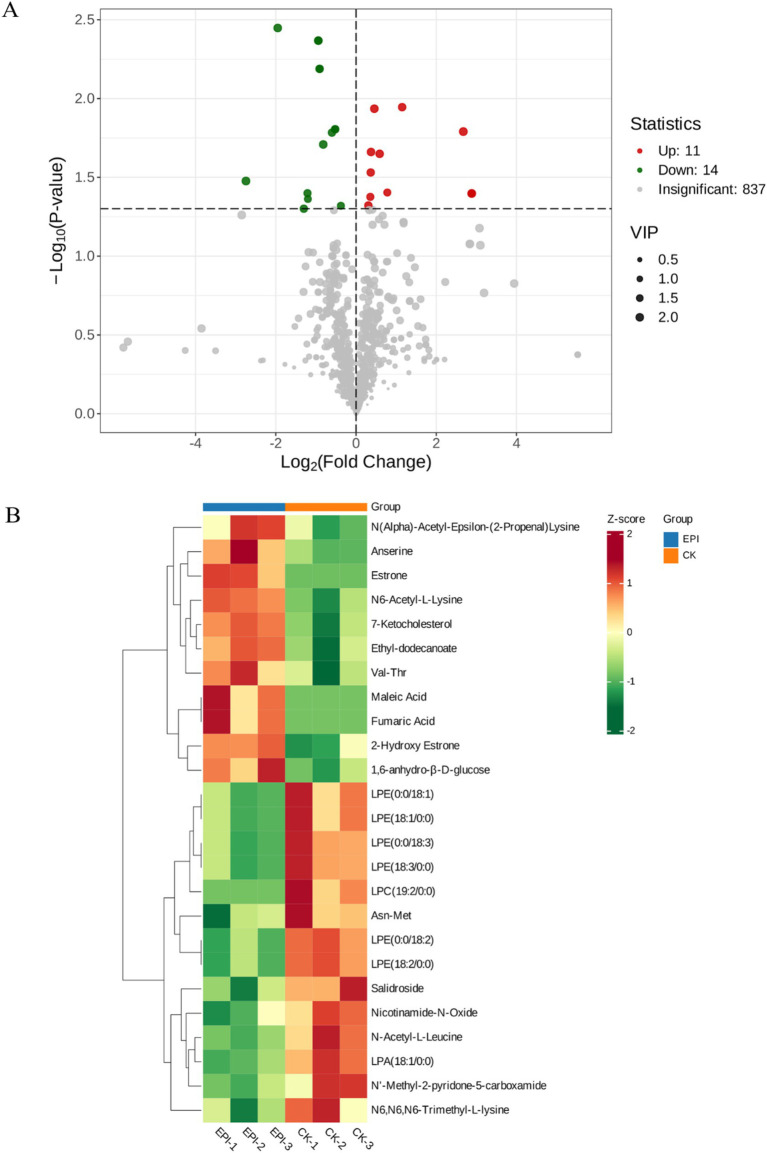
Volcano plot of intergroup differential metabolites **(A)**. The red represents significantly upregulated metabolites, green represents significantly downregulated metabolites, and gray represents non-significantly changed metabolites. The size of the dots represents the VIP value. Heatmap of clustering analysis of gut differential metabolites **(B)**. The color blocks at different positions represent the relative expression levels of the corresponding metabolites at those locations. CK, control group; EPI, *Epimedium* group.

Through a combination of univariate statistical analysis and multivariate statistical analysis methods, we screened for differential metabolites between the groups. The screening criteria used were a *p*-value <0.05 & VIP ≥ 1. The results of the screening were visualized in a volcano plot, revealing a total of 25 differential metabolites, with 11 metabolites being upregulated and 14 metabolites being downregulated (Figure 6A). In addition, hierarchical clustering analysis of the differential metabolites showed that the serum differential metabolites were distinct between the groups, as shown in Figure 6B. Red indicates an increase in the relative content of substances, while green indicates a decrease in the relative content of substances. The intra-group clustering of the CK group and the EPI group was ideal. Compared with the CK group, the expression levels of anserine, 2-hydroxy estrone, and estrone were upregulated in the EPI group, while the expression levels of nicotinamide-n-oxide, asn-met, and salidroside were downregulated (Table 4). Dietary supplementation with *Epimedium* led to significant changes in the serum metabolism of the Chinese forest musk deer.

**Table 4:** Partial information on differential metabolites.

Name	Vip	*p*-value	Up/Down	Cas
Ethyl-dodecanoate	1.91	0.029	Up	106-33-2
N6, N6, N6-Trimethyl-L-lysine	1.71	0.048	Down	–
7-Ketocholesterol	2.01	0.022	Up	566-28-9
Nicotinamide-N-Oxide	1.80	0.039	Down	1986-81-8
Anserine	2.02	0.022	Up	584-85-0
2-Hydroxy Estrone	1.92	0.047	Up	362-06-1
N(Alpha)-Acetyl-Epsilon-(2-Propenal) Lysine	1.77	0.042	Up	99124-74-0
Asn-Met	1.65	0.043	Down	–
Salidroside	1.89	0.016	Down	10338-51-9
Estrone	2.16	0.016	Up	53-16-7

Vip, variable importance in projection; Up/Down, levels of metabolites adjusted upward/downward; Cas, Chemical Abstracts Service.

#### 3.3.3 Serum metabolic pathways

**Figure 7:**
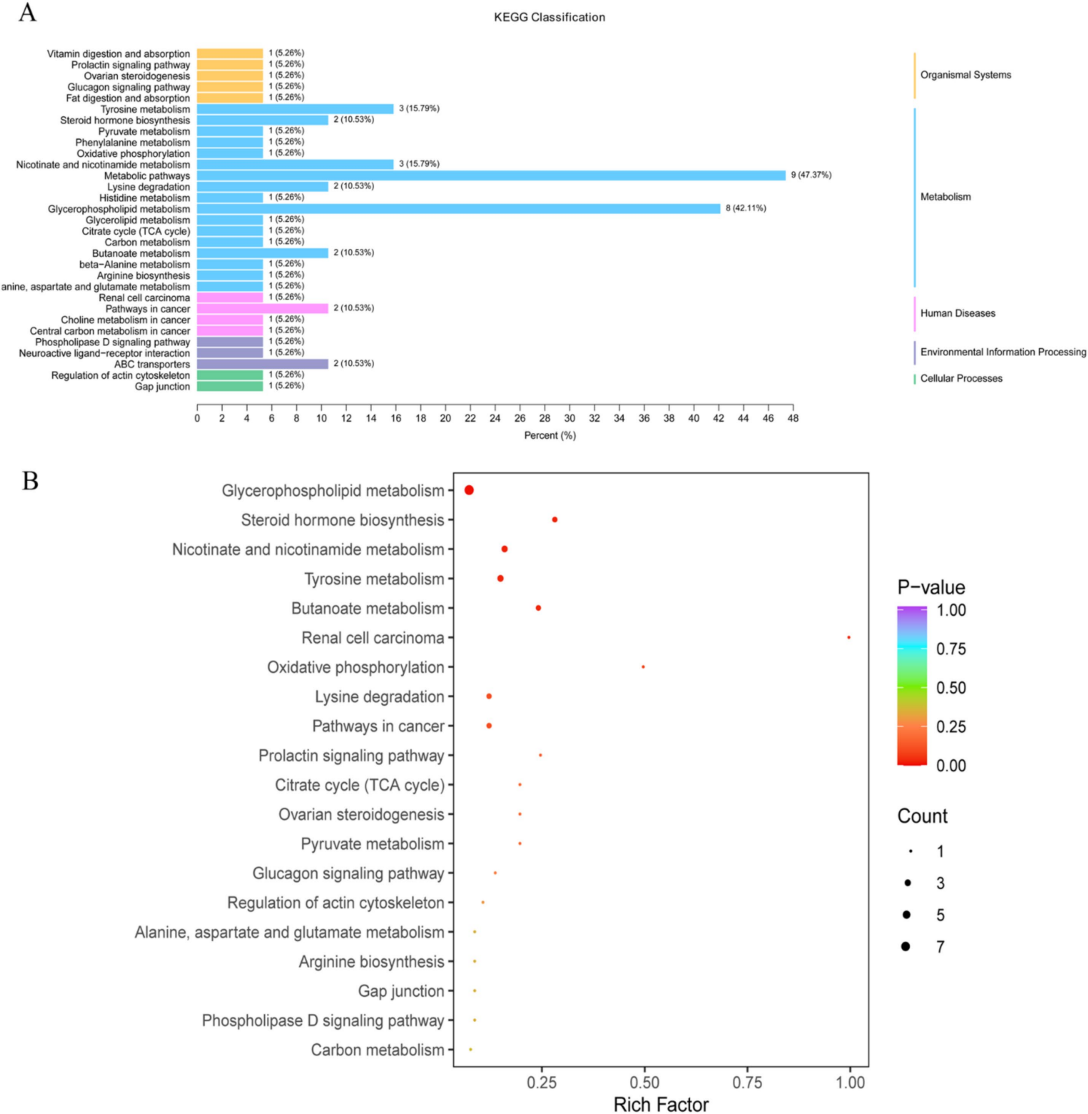
Differential metabolite pathway classification diagram **(A)**. The color represents the *p*-value of the enrichment analysis, with a deeper red color indicating a more significant degree of enrichment. Enrichment plot of differential metabolite pathways **(B)**. The color of the bubble represents the *p*-value of the enrichment analysis, with a deeper red color indicating a more significant degree of enrichment. The size of the dots represents the number of differential metabolites enriched in that pathway. CK, control group; EPI, *Epimedium* group.

Based on the differential metabolites identified, KEGG pathway enrichment analysis was performed, and the enriched pathways were visually represented in bar charts and bubble plots. The pathways with significantly different metabolisms were selected based on the significance index *p*-value. The KEGG results showed that the differential metabolites between the CK and EPI groups were annotated into five categories of metabolic pathways, with metabolic pathways and glycerophospholipid metabolism being the most significantly enriched (Figure 7A). Additionally, KEGG pathway enrichment analysis indicated that the major metabolic pathways altered in the EPI group compared to the CK group included glycerophospholipid metabolism, tyrosine metabolism, nicotinate and nicotinamide metabolism, ovarian steroidogenesis, and beta-alanine metabolism (Figure 7B). Among these, glycerophospholipid metabolism and tyrosine metabolism were the most significantly altered pathways, suggesting that the supplementation of *Epimedium* might have had a notable impact on metabolic pathways related to lipid metabolism and hormone regulation.

### 3.4 Correlation analysis for differential microbes and metabolites

**Figure 8:**
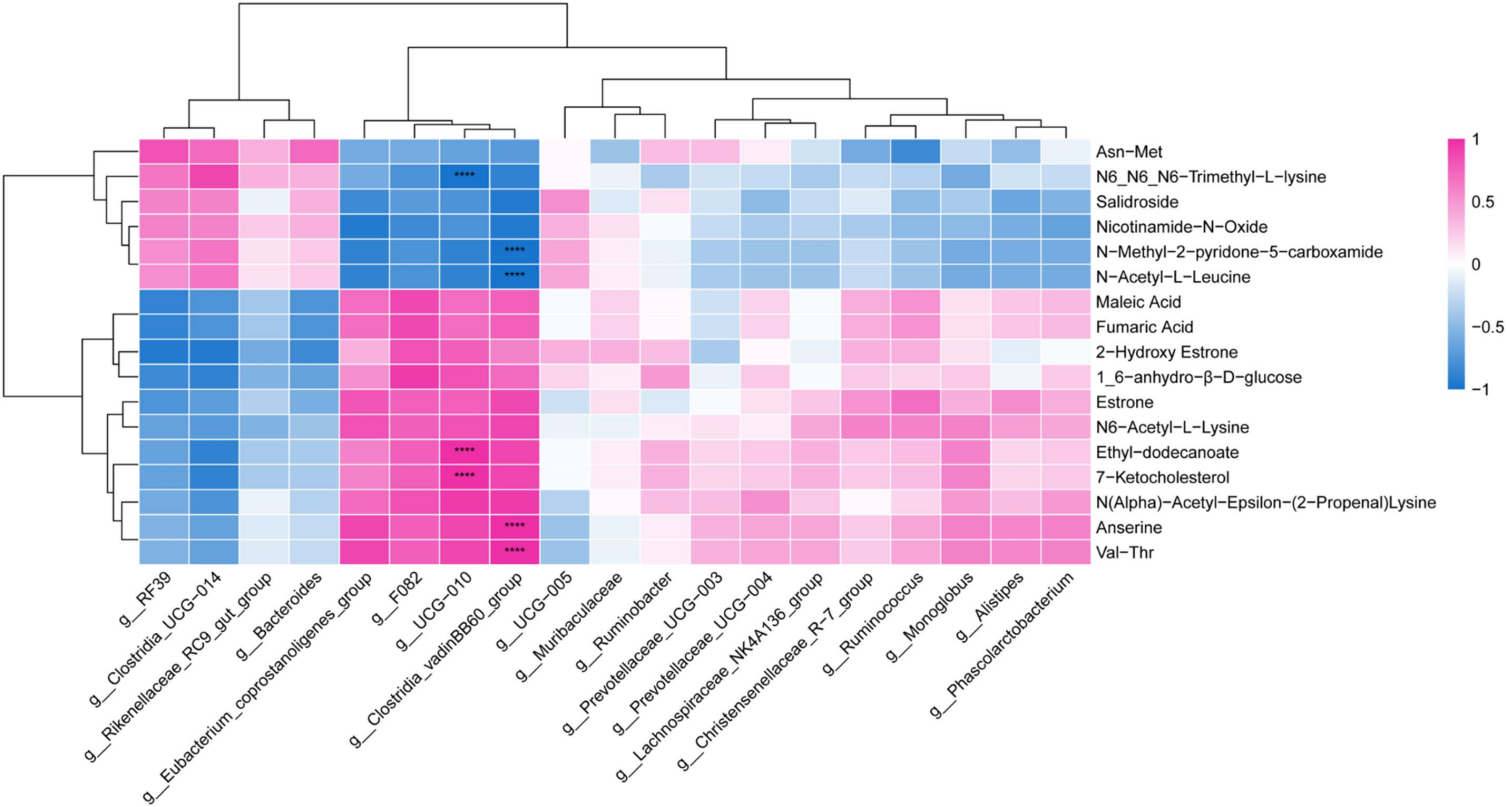
Microbiome-metabolome correlation heatmap. **** *p* < 0.0001. Red represents a positive correlation, and blue represents a negative correlation.

Through Pearson correlation analysis, it was found that there was a correlation between differential metabolites and differential bacterial genera. As shown in Figure 8, the *Clostridia_vadinBB60_group* exhibits a positive correlation with anserine and N(alpha)-acetyl-epsilon-(2-propenal) lysine, and a negative correlation with N-acetyl-L-leucine and N-methyl-2-pyridone-5-carboxamide (*p* < 0.05). Similarly, *UCG-010* shows a positive correlation with 7-ketocholesterol and ethyl-dodecanoate, while displaying a negative correlation with N6, N6, N6-trimethyl-l-lysine (*p* < 0.05).

## 4 Discussion

*Epimedium* possesses various bioactive properties, and its appropriate supplementation as a feed additive can effectively enhance feed utilization rates in animals, thereby significantly improving economic benefits (32). In this study, compared to the CK group, dietary supplementation with *Epimedium* increased feed intake and feed conversion ratio in the experimental group, with a trend towards increased ADG. Zhang et al. (21) found that adding *Epimedium* to the diet improved intestinal function and gut microbiota in broilers, significantly increasing ADG and enhancing growth performance. This effect is attributed to the flavonoids in *Epimedium*, which can promote the secretion of digestive enzymes, enhancing the digestion and absorption of nutrients in the feed, thereby improving feed efficiency and promoting animal growth (33, 34). *Epimedium* leaves can enhance testosterone production in rat Leydig cells by regulating the expression of steroidogenic enzymes (35). Research has shown that polyphenolic compounds in *Epimedium* significantly elevate levels of reproductive hormones such as testosterone and luteinizing hormone in albino rats, while significantly reducing levels of progesterone and estradiol (36). However, in our study, we found that adding *Epimedium* to the diet increased fecal testosterone levels, but had no effect on progesterone and estradiol levels. This may be due to the unique digestive system and physiological characteristics of musk deer, which make their metabolic mechanisms significantly different from those of mice. Additionally, the experimental conditions in our study (such as housing environment, diet, sample processing, etc.) may differ from those in mouse experiments. These differences in conditions may have affected the measurement of progesterone levels. These findings suggest that *Epimedium* has a positive impact on increasing feed intake and regulating hormone levels in the Chinese forest musk deer, thereby effectively maintaining overall health. The yield of musk is closely related to fluctuations in hormone levels (14). Studies have indicated that quercetin can promote musk secretion by regulating the hormone production (such as testosterone and estradiol) in the Chinese forest musk deer (37). Additionally, administering exogenous testosterone during the non-secretory season can also promote musk secretion in the Chinese forest musk deer (38, 39). In this study, the level of testosterone increased after the addition of *Epimedium*, hypothesizing that *Epimedium* may increase musk yield by influencing hormone levels. Future studies should consider to provide a more comprehensive assessment of the benefits of *Epimedium* supplementation in the Chinese forest musk deer.

The diversity of gut microbiota is associated with the host’s health status, and *Epimedium* can effectively improve animal health by modulating the composition of the gut microbiota (40). In this study, after adding *Epimedium* to the Chinese forest musk deer diet, the Chao1 index, observed species index, and Shannon index in the EPI group were all higher than in the CK group, but these differences did not reach statistical significance. This may indicate that the addition of *Epimedium* has a positive effect on the richness and diversity of the Chinese forest musk deer gut microbiota, but this effect was not detected significantly due to the limited sample size or experimental conditions. According to the annotation results, the main bacterial phyla in the gut of the Chinese forest musk deer are Bacteroidetes, Firmicutes, and Proteobacteria, with Firmicutes being the dominant phylum, which is consistent with previous research findings (41, 42). Firmicutes is one of the main microbiota in the gut of ruminants, capable of producing short-chain fatty acids (SCFAs) such as acetic acid, propionic acid, and butyric acid through fermentation (43). These SCFAs play a crucial role in maintaining gut health and amino acid metabolism. Additionally, ecological biotherapy strategies enriched with Firmicutes can be used to prevent or treat colitis, demonstrating potential anti-inflammatory properties (44). In contrast, Proteobacteria are considered potentially harmful microbiota, as their increased relative abundance is associated with various gut diseases and inflammatory bowel diseases (45). They are present in lower amounts in the gut of healthy animals (46). Abnormal growth of Proteobacteria may represent an imbalance in the gut microbiota and could serve as a potential marker for disease risk (47). After adding *Epimedium* to the Chinese forest musk deer diet, the relative abundance of Firmicutes in the EPI group was higher than in the control group, while the relative abundance of Proteobacteria was lower. This indicates that *Epimedium* has a positive effect on regulating the composition of the musk deer gut microbiota. At the genus level, the dominant microbiota in the gut is *Bacteroides*. We found that the relative abundance of *Bacteroides* in the EPI group was higher than that in the CK group. Studies have demonstrated that an increase in the abundance of *Bacteroides* species can reduce lipopolysaccharide (LPS) levels and inhibit immune responses, thereby preventing the onset of cardiovascular diseases in humans (48). *Bacteroides* can effectively degrade polysaccharides, providing essential nutrients for other gut microbiota, which promotes symbiotic relationships and synergistic interactions among gut microbiota (49). In conclusion, we hypothesize that *Epimedium* has potential benefits in regulating the gut microbiota of the Chinese forest musk deer by increasing the abundance of beneficial bacteria and reducing the abundance of harmful bacteria, thereby maintaining the stability of the gut microbiome composition. Although we minimized the impact of parasites on the gut microbiota of the Chinese forest musk deer through regular health checks and preventive treatment with anti-parasitic drugs, recent studies have shown that parasitic infections can affect the composition and function of the gut microbiota (50). These findings suggest that future research should further investigate the interaction between parasitic infections and the gut microbiota to achieve a more comprehensive understanding of the various factors influencing the health of the Chinese forest musk deer.

In this study, the overall composition of serum metabolites showed differences. A total of 25 differential metabolites were detected in the serum. We found that dietary supplementation with *Epimedium* may impact metabolic pathways involved in lipid metabolism, antioxidation, and hormone regulation. *Epimedium* is believed to help increase sex hormone levels and effectively improve sexual function (51). Fluctuations in sex hormone levels can affect the estrous cycle and reproductive capacity of animals (52). Estrone may indirectly influence the behavior of the male Chinese forest musk deer during the breeding season, promoting musk secretion (53). Additionally, sex hormones such as testosterone, estrogen, and cortisol play a significant role in determining the composition of musk (39). In this experiment, compared to the CK group, the levels of estrone and 2-hydroxy estrone in the EPI group increased, which positively impacted the ovarian steroidogenesis and prolactin signaling pathways. Anserine, a natural compound found in animal tissues, has been shown to effectively reduce oxidative stress-induced damage by regulating antioxidant-related signaling pathways such as Keap1-Nrf2 and JNK-Caspase-3, thereby exerting antioxidant effects (54, 55). Chen et al. (56) found that anserine can increase the activity of serum superoxide dismutase and reduce levels of malondialdehyde, alkaline phosphatase, and alanine aminotransferase, thereby alleviating hyperuricemia in rats. In this experiment, anserine levels increased and were enriched in histidine metabolism and beta-alanine metabolism pathways. Histidine metabolism, a critical amino acid metabolic process, plays a vital role in protein synthesis, neurotransmitter function, and the immune system, and has a positive impact on appetite regulation (57, 58). *β-*Alanine can combine with L-histidine to form carnosine, a compound with antioxidant properties that can neutralize free radicals and reduce oxidative stress-induced damage to muscle cells (59, 60). Glycerophospholipid metabolism involves various enzymes and metabolic pathways that regulate lipid synthesis and degradation, thereby influencing cellular lipid balance and metabolism. The products of glycerophospholipid metabolism can affect the body’s immune response by modulating the synthesis and release of inflammatory mediators (61). In summary, *Epimedium* may positively influences related metabolic pathways by regulating the production of key metabolites, effectively increasing hormone levels and antioxidant capacity. This contributes to the stabilization of metabolic and physiological functions in the Chinese forest musk deer.

Correlation analysis revealed associations between *Clostridia_vadinBB60_group* and *UCG-010* with different metabolites. We found that *Clostridia_vadinBB60_group* was positively correlated with anserine. As a beneficial bacterium, *Clostridia_vadinBB60_group* can effectively degrade complex organic compounds such as cellulose, hemicellulose, and polysaccharides, producing SCFAs like acetate, butyrate, and propionate, which play a crucial role in host energy supply, gut health, and microbial balance (62–64). This is particularly important in ruminant production, where it contributes to improving feed conversion efficiency (65). Dietary supplementation of *Epimedium*, the level of anserine increased, which may be related to the increased abundance of *Clostridia_vadinBB60_group* in this study. Further experiments are needed to verify this hypothesis by vitro culture and mechanistic studies. *UCG-010* is widely present in the intestines of animals, particularly within the digestive systems of ruminants, where it plays a significant role in the gut microbiota by breaking down complex carbohydrates, thereby effectively promoting nutrient absorption (66, 67). In this study, *UCG-010* was found to be positively correlated with 7-ketocholesterol. After adding the diet with *Epimedium*, the relative abundance of *UCG-010* increased, which may affect the metabolism of 7-ketocholesterol, predicting it may affect the metabolism of 7-ketocholesterol. Further research is needed to focus on clarifying their regulation relationship and systematic mechanism.

In this study, only a small number of the Chinese forest musk deer samples (*n* = 3) were used for growth performance and metabolomics analysis, which is directly related to the rarity and protected status of this species. The limited number of animals included in our study may introduce biases. Specifically, the small sample size may not fully capture the natural variability within the species, potentially affecting the robustness of our statistical analyses. Due to insufficient statistical power, true biological effects may be overlooked. Furthermore, the limited representation of the Chinese forest musk deer population may not adequately reflect the complexity and diversity of their biological systems, leading to an incomplete understanding of the underlying mechanisms. To address these limitations, future studies should aim to replicate our findings with larger and more diverse sample sizes. This would enhance the reliability and validity of the results, allowing for a more comprehensive exploration of the relevant biological pathways. However, given the conservation status and ethical considerations associated with studying the Chinese forest musk deer, alternative approaches such as non-invasive sampling methods or collaborations with multiple research institutions may be necessary to effectively increase the sample size.

## 5 Conclusion

Dietary supplementation with *Epimedium* may increase the feed intake and feed conversion ratio of the Chinese forest musk deer, and positively influence their hormone levels, particularly with an observed increase in testosterone levels. Additionally, *Epimedium* may enhance the richness and diversity of the gut microbiota in the Chinese forest musk deer and altered serum metabolism, including amino acid metabolism, lipid metabolism, and energy metabolism. In summary, appropriate supplementation of *Epimediu*m may contribute to the improvement of hormone levels, gut microbiota, and serum metabolite composition in the Chinese forest musk deer.

## Data Availability

The data presented in the study are deposited in the NCBI repository, accession number PRJNA1195406.

## Footnotes

^1^ https://hmdb.ca/

^2^ https://metlin.scripps.edu/

^3^ http://www.kegg.jp/kegg/compound/

^4^ http://www.kegg.jp/kegg/pathway.html
